# An Interpretable and Expandable Deep Learning Diagnostic System for Multiple Ocular Diseases: Qualitative Study

**DOI:** 10.2196/11144

**Published:** 2018-11-14

**Authors:** Kai Zhang, Xiyang Liu, Fan Liu, Lin He, Lei Zhang, Yahan Yang, Wangting Li, Shuai Wang, Lin Liu, Zhenzhen Liu, Xiaohang Wu, Haotian Lin

**Affiliations:** 1 School of Computer Science and Technology Xidian University Xi'an China; 2 State Key Laboratory of Ophthalmology Zhongshan Ophthalmic Center Sun Yat-sen University Guangzhou China; 3 School of Software Xidian University Xi'an China; 4 Institute of Software Engineering Xidian University Xi'an China

**Keywords:** deep learning, object localization, multiple ocular diseases, interpretable and expandable diagnosis framework, making medical decisions

## Abstract

**Background:**

Although artificial intelligence performs promisingly in medicine, few automatic disease diagnosis platforms can clearly explain why a specific medical decision is made.

**Objective:**

We aimed to devise and develop an interpretable and expandable diagnosis framework for automatically diagnosing multiple ocular diseases and providing treatment recommendations for the particular illness of a specific patient.

**Methods:**

As the diagnosis of ocular diseases highly depends on observing medical images, we chose ophthalmic images as research material. All medical images were labeled to 4 types of diseases or normal (total 5 classes); each image was decomposed into different parts according to the anatomical knowledge and then annotated. This process yields the positions and primary information on different anatomical parts and foci observed in medical images, thereby bridging the gap between medical image and diagnostic process. Next, we applied images and the information produced during the annotation process to implement an interpretable and expandable automatic diagnostic framework with deep learning.

**Results:**

This diagnosis framework comprises 4 stages. The first stage identifies the type of disease (identification accuracy, 93%). The second stage localizes the anatomical parts and foci of the eye (localization accuracy: images under natural light without fluorescein sodium eye drops, 82%; images under cobalt blue light or natural light with fluorescein sodium eye drops, 90%). The third stage carefully classifies the specific condition of each anatomical part or focus with the result from the second stage (average accuracy for multiple classification problems, 79%-98%). The last stage provides treatment advice according to medical experience and artificial intelligence, which is merely involved with pterygium (accuracy, >95%). Based on this, we developed a telemedical system that can show detailed reasons for a particular diagnosis to doctors and patients to help doctors with medical decision making. This system can carefully analyze medical images and provide treatment advices according to the analysis results and consultation between a doctor and a patient.

**Conclusions:**

The interpretable and expandable medical artificial intelligence platform was successfully built; this system can identify the disease, distinguish different anatomical parts and foci, discern the diagnostic information relevant to the diagnosis of diseases, and provide treatment suggestions. During this process, the whole diagnostic flow becomes clear and understandable to both doctors and their patients. Moreover, other diseases can be seamlessly integrated into this system without any influence on existing modules or diseases. Furthermore, this framework can assist in the clinical training of junior doctors. Owing to the rare high-grade medical resource, it is impossible that everyone receives high-quality professional diagnosis and treatment service. This framework can not only be applied in hospitals with insufficient medical resources to decrease the pressure on experienced doctors but also deployed in remote areas to help doctors diagnose common ocular diseases.

## Introduction

Although there have been many artificial intelligence-based automatic diagnostic platforms, the diagnostic results produced by such computer systems cannot be easily understood. Artificial intelligence that obtains diagnostic results from the computational perspective cannot provide the reason that is depicted as clinical practice for a given diagnosis. Some researchers have attempted to make the conclusion obtained from artificial intelligence methods explainable, such as Raccuglia et al used a decision tree to understand the classification result from the support vector machine [1]. Hazlett et al used a deep belief network, a reverse trackable neural network, to find diagnostic evidence of autism [2]. Zhou et al used the output of the last full-connected layer of the convolution neural network to infer which part of an image causes the final classification result, which also provides the evidence of classification [3]. In addition, Zeiler et al used occlusion test to study which parts of images produce a given classification result [4]. These studies made great achievements in explainable artificial intelligence, but readily explainable automatic diagnostic systems are still rare. The primary cause is that these explainable methods did not explain their result according to human thought patterns. Therefore, this research aims to make additional progress based on previous studies.

There are many existing works about the automatic diagnosis of different types of diseases with medical imaging, but all these works are isolated; those cannot regard all diseases shown in a specific format of medical images with a unified perspective, which is common in natural image processing and practical medical scenes. On the other hand, once all diseases are regarded as unified, the extensibility for integrating other types of medical imaging or disease will be easy. The diagnosis of ophthalmic diseases is highly dependent on observing medical images, so this work selected ophthalmic images that represent multiple ocular diseases as material and treated them with a consistent view. Of note, the unified automatic diagnostic procedure is the simulation of the work flow of doctors. An explainable artificial intelligence-based automatic diagnosis platform offers many advantages. First, it can increase the confidence in the diagnostic results. Second, it assists doctors to perfect the diagnosing thinking. Third, it helps medical students deepen the medical knowledge. Finally, it can clear a path toward diagnosing higher numbers of diseases from a unified perspective.

Besides, doctors can diagnose diseases by observing medical images, but doctors from many specialties and subspecialties cannot tackle all diseases. If a patient suffers from more than one type of disease, the system can tackle these diseases simultaneously. This work plans to integrate the experience of doctors from many subspecialties to construct an omnipotent ophthalmologist.

Thus, to create an explainable automatic diagnostic system with artificial intelligence, we simulated the workflow of doctors to help artificial intelligence follow the patterns of human thought. This research aims to apply artificial intelligence techniques to fully simulate the diagnostic process of doctors so that reasons for a given diagnosis can be illustrated directly to doctors and patients.

In this research, we designed an interpretable and expandable framework for multiple ocular diseases. There are 4 stages in this diagnostic framework: primary classification of disease, detection of each anatomical parts and foci, judging the conditions of anatomical parts, and foci and providing treatment recommendations. The accuracies of all stages surpass 93%, 82%-87%, 79%-98%, and 95%, respectively. Not only is this system an interpretable diagnostic tool for doctors and patients but it also facilitates the accumulation of medical knowledge for medical students. Moreover, this system can be enriched to cover more ophthalmic diseases or more diseases of other specialties to provide more services as the workflow of doctors. Telemedicine [5] can combine medical experts and patients with considerable low cost. This research develops an interpretable and expandable telemedical artificial intelligence diagnostic system, which can also effectively improve the undesirable condition that medical resource with high quality is not adequate and the distribution of it is not even. Finally, the health level of people all over the world and the medical condition of underdeveloped countries can be improved with the help of a computer network.

## Methods

### Data Preparation

Data are important for data-driven research [6]. The dataset is examined by all members of our team. Besides, we developed some programs to facilitate the examination of data. All images were collected in the Sun Yat-sen University Zhongshan Ophthalmic Center, which is the leading ophthalmic hospital in China [7]. In order to simulate the experience and diagnostic process of doctors, all images were segmented into several parts according to anatomical knowledge or diagnostic experiences and, then, were annotated. Next, multiple attributes of all parts were classified as the actual states of these parts (including foci). All the relevant aspects of the data (images, coordinates of each part, and the attribute information) were used to train an artificial intelligence system. This data preparation process can not only help simulate the diagnostic process of doctors but also facilitate many follow-up studies such as medical image segmentation, clinical experience mining, and integration of refined diagnosing of multiple diseases.

**Figure 1:**
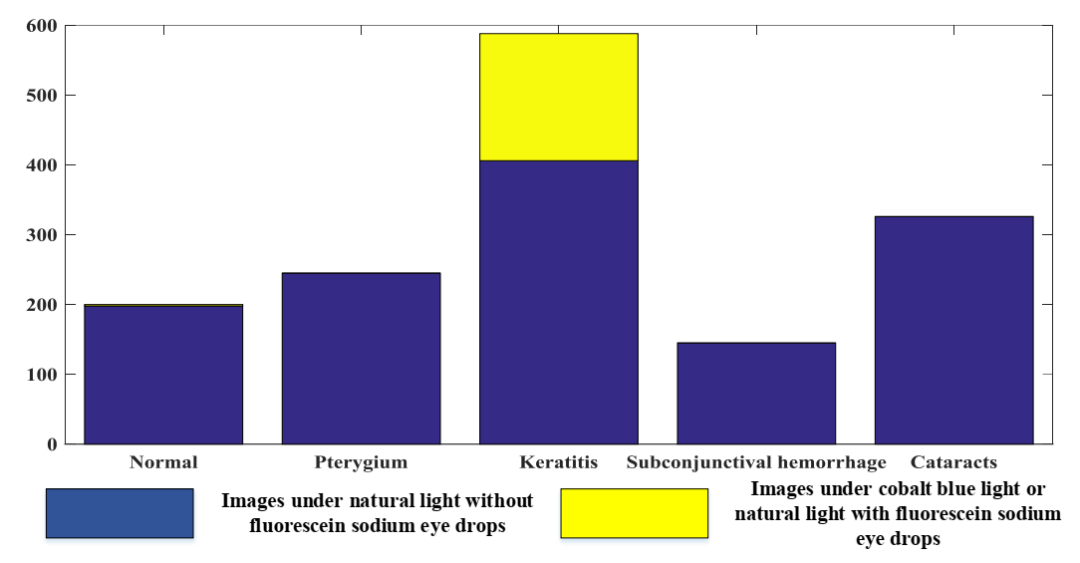
Information of image dataset.

**Figure 2:**
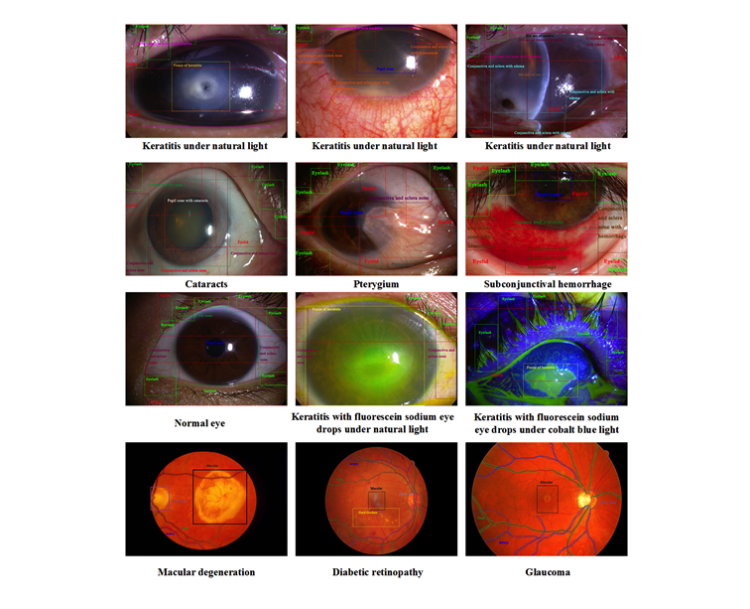
Examples of each object in terms of each type of disease or normal eye.

We collected 1513 images that can be classified into 5 classes (normal, pterygium [9], keratitis [10], subconjunctival hemorrhage [11], and cataract [12]). Figure 1 lists the number of images of each class. Furthermore, the examples of objects to be detected in images are shown in Figure 2; for fundus images (the last row), the localized objects include an artery (blue), vein (green), the macula (black), the optic disc (light purple), hard exudate (yellow), and so on. For other types of images, the objects to be localized include the eyelid (red), eyelash (green), keratitis focus (yellow), cornea and iris zone with keratitis (pink), the pupil zone (blue), conjunctiva and sclera zone with hyperemia (orange), the conjunctiva and sclera zone with edema (light blue), the conjunctiva and sclera zone with hemorrhage (brown), the pupil zone with cataracts (white), the slit arc of the cornea (black), cornea and iris zone (dark green), the conjunctiva and sclera zone (purple), pterygium (gray), the slit arc of keratitis focus (dark red), and the slit arc of the iris (light brown). Table 1 lists the detailed diagnostic attributes to be classified, and each diagnostic information corresponds to a classification problem. The diagnostic information in Table 1 is corresponding to stage 3 (see Methods). This information is essential and fundamental for diagnosing and providing treatment advice and will be determined in stage 3 of the interpretable artificial intelligence system (see Methods). All information (object annotation and diagnostic information) was double-blind marked by the annotation team, which consisted of 5 experienced ophthalmic doctors and 20 medical students. The annotation of fundus images was completed; however, the experiments on fundus images were not finished. Because of the intrinsic characteristics of the fundus image, the output of the annotation method for fundus image is suitable for semantic segmentation.

### Methodology

**Figure 3:**
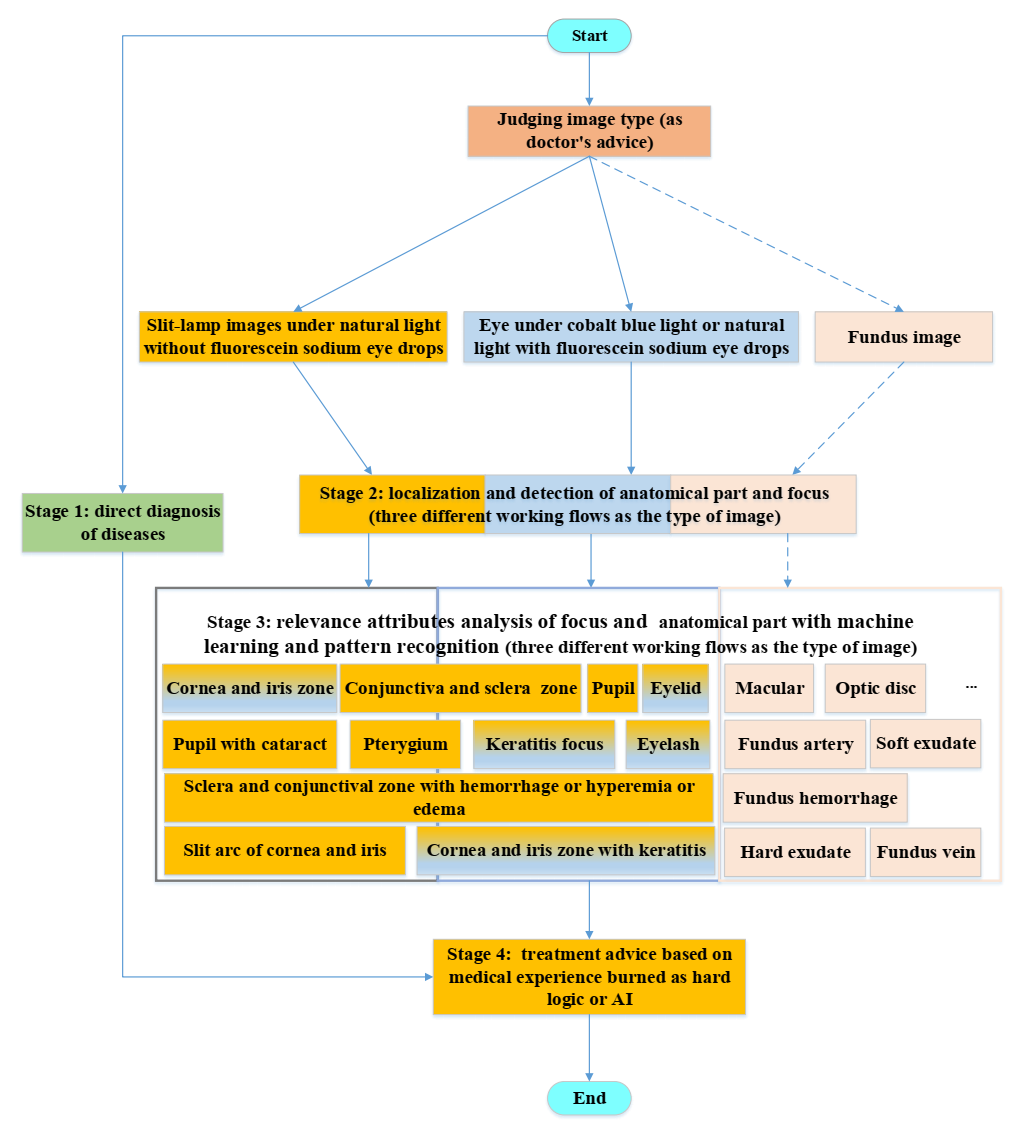
Architecture of the overall framework for interpretable diagnosis of multiple ocular diseases. AI: artificial intelligence.

The framework consists of 4 functional stages as follows: (1) judging the class of disease, preliminary diagnosis that is completed with original image without any processing; (2) detecting each part of image, localization of anatomical parts, and foci that are used to discern different parts with different appearance so that more careful checking can be guaranteed; (3) classifying the attributes of each part, severity and illness assessment, which is closely connected to the second stage, is used to determine the condition of the illness; and (4) providing treatment advice according to the results from the first, second, and third stages, except for the treatment advice of a pterygium is from artificial intelligence, whereas treatment advice of other diseases is from experiences of doctors. First, the disease is primarily identified during stage 1. Second, all anatomical parts and foci are localized during stage 2, and important parts (cornea and iris zone with keratitis and pterygium) are segmented for the analysis in stage 3. Then, the attributes of all anatomical parts and foci are determined during stage 3. Then, the treatment advice is provided in stage 4. The whole process imitates the diagnostic procedure of doctors so that the reasons for a given diagnosis can be tracked and used to construct an evidence-based diagnostic report. Finally, treatment advice can be provided according to the full workflow presented above. Figure 3 shows the flowchart of this system. The analysis of fundus images is coming soon and will be easily integrated into this system quickly as the same idea with existing images. The first, second, and third function is fully based on artificial intelligence, which is trained with dataset; the fourth function is dependent on both artificial intelligence and the experience of doctors.

**Table 1:** Detailed diagnostic information regarding the dataset.

Disease	Diagnostic information (Number of classification problems)	Values of diagnostic information	Type of image
Pterygium	Whether the body of the pterygium is hypertrophied Whether pseudo pterygium is present Whether the head of the pterygium is uplifted Whether the head and body of the pterygium is hyperemic Whether the pterygium is in the progressive period	Yes or no	Images under natural light without fluorescein sodium eye drops
Keratitis	Turbidity degree of the cornea Stage of keratitis Corneal neovascularization Edge of foci is clear The condition of illness based on dyeing	Pupil zone is invaded by turbidity or not Infiltration stage and ulcer stage, perforation stage, or convalescence Yes or no No dyeing and dot staining, sheet dyeing, or dyeing with coloboma	Images under cobalt blue light or natural light with fluorescein sodium eye drops [8]

Machine learning, especially deep learning technique represented by the convolutional neural network (CNN), is becoming the effective computer vision tool for automatically diagnosing diseases using biomedical images. It has been widely applied in the medical image classification and automatic diagnosis of disease, such as the diagnosis of attention deficit hyperactivity disorder with functional magnetic resonance imaging [13]; gradation of brain tumor [14], breast cancer [15], and lung cancer [16]; and diagnosis of skin disease [17], kidney disease [18], and ophthalmic diseases [19-23]. In this research, inception_v4 [24] and residual network (Resnet) [25] (101 layers) were used to carry out stage 1 and stages 3 and 4, respectively. While stage 1 (inception_v4) can give a general diagnostic conclusion, stages 3 (Resnet) and 4 (Resnet) can provide further information about diseases and treatment recommendations. In this research, cost-sensitive CNN was adopted because the imbalanced classification is common in this research. Inception_v4 is a wider and deeper CNN that is suitable for careful classification (the difference between all classes is easily neglected sometimes). Resnet is a type of thin CNN, the architecture of which is full of cross-layer connections. The objective function is transformed to fit the residual function so that the performance of Resnet is improved considerably. In addition, Resnet is suitable for rough classification (the difference between all classes does not need to be carefully analyzed). In addition, we chose Resnet with 101 layers whose volume is adequate for the classification problems in this research. Stage 1 is a 5-classes classification, with some classes being very similar in color and shape; thus, inception_v4 is chosen in stage 1. As other classification problems are limited in one specific disease, Resnet is selected in stages 3 and 4. Furthermore, the chain rule of derivatives based on the stochastic gradient descent algorithm [26] was used to minimize the loss function.

Faster-region based convolutional neural network (RCNN), an effective and efficiency approach, was adopted to localize the anatomical parts and foci (Stage 2). Faster-RCNN [27] is developed on the basis of RCNN [28] and Fast-RCNN [29], which originally applied superpixel segmentation algorithm to produce proposal regions, whereas Faster-RCNN uses an anchor mechanism to generate region proposals quickly and then adopts 2-stage training to obtain the transformations of bounding box repressor and classifier. The first stage of Faster-RCNN is region proposal network, which is responsible for generating region proposals. Then, whether the proposals are objects or not are judged, and the coordinates of each object are primary regressed. The second stage is judging the class of each object and eventually regressing the coordinate of each object, which is the same as RCNN and Fast-RCNN. In this research, pretrained ZF (Zeiler and Fergus [4]) network was exploited to save training time.

### Experimental Settings

This system was implemented with convolutional architecture for fast feature embedding [30] (Berkeley Vision and Learning Center deep learning framework) and Tensorflow [31]; all models were trained in parallel on four NVIDIA TITAN X GPUs. For the classification problem, indicators applied to evaluate the performance are as follows:

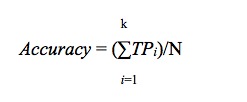

Precisioni= TPi/(TPi+ FPi

TPi+ FPi

SensitivityiTPR, RecallTPi/(TPi+ FNi

TPi+ FNi

FNRifalse-negative rateFNiTPFNi

TP + FNi

Specificityi= TNi/TNi+ FPi

TNi+ FPi

FPRi(false-positive rate) = FPi/TNiFPi

TNi + FPi

where *N* is the total number of samples; *P*_*i*_ indicates the number of correctly classified samples of *i* th class; *k* is the number of classes in specific classification problem;*TP*_*i*_ denotes the number of samples that are correctly classified as *i* th class; *FP*_*i*_ is the number of samples that are wrongly recognized as *i* th class; *FN*_*i*_ denotes the number of samples that are classified as *j* th class, *j* ϵ [1,c]/*i*; *TN*_*i*_ is the number of samples recognized as negative *j* th class, *j* ϵ [1,c]/*i*. All the above performance indicators can be computed with a confusion matrix. In addition, the receiver operating characteristics (ROC) curve, which indicates how many samples of *i* th class are recognized conditioned on a specific number of *j* th class (*j* ϵ [1,c]/*i*), are classified as *i* th class, PR (precision recall) curve, which illustrates how many samples of *j* th class are recognized as samples of *i* th class conditioned on a specific number of *j* th class (*j* ϵ [1,c]/*i*), are classified as *i* th class and area under the ROC curve (AUC), which means the area of the zone under the ROC curve was also adopted to assess the performance [32]. The indicators (precision, sensitivity, specificity, ROC curve with AUC, and PR curve) were only used to evaluate the performance of binary classification problems. Furthermore, accuracy and confusion matrix were used to evaluate the performance of multiclass classification problems.

For object localization problem, the interpolated average precision is always used to evaluate the performance [33]. The interpolated average precision is computed with the PR curve using the equation presented below:

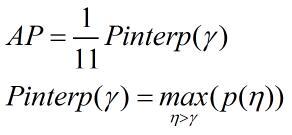



In the equation, p(η) is the measured precision at specific recall η. In this research, 4-fold cross-validation was used to evaluate the performance of this system firmly for all classification problems and localization problems. The application of the cost-sensitive CNN is dependent on the distribution of the dataset in specific classification problems. Except for the classification problems 1, 6, and 8, other classification problems in stages 3 and 4 were completed with the cost-sensitive CNN.

## Results

### Performance of Stages 1 and 2

**Figure 4:**
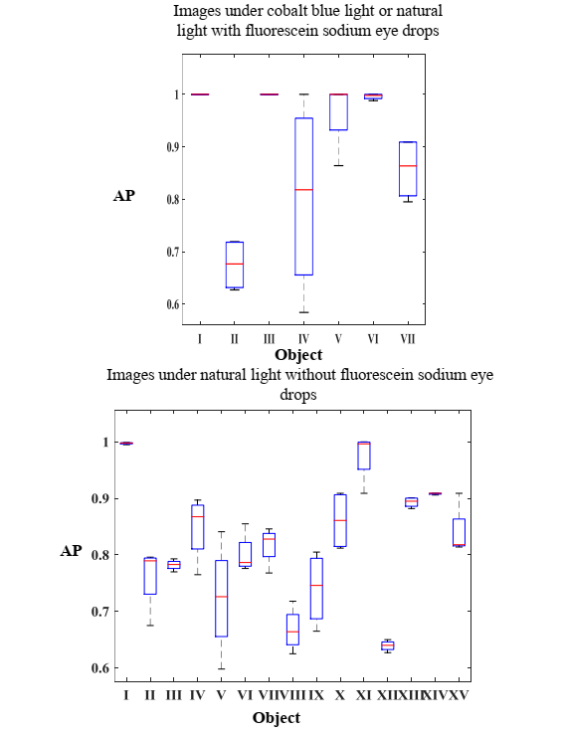
Performance of stage 1.

**Figure 5:**
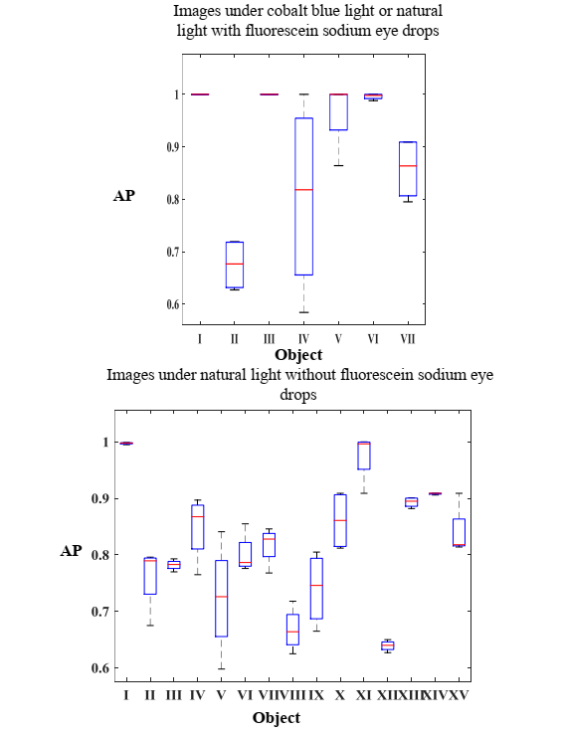
Performance of stage 2. AP: average precision.

All stages and the whole work flow of this system were completed with acceptable performance. The 4 stages in the framework were separately trained and validated, and all relevant results in stages 1 and 2 are shown in Figures 4 and 5. The rows and columns of all heat maps stand for ground truth labels and predicted labels, respectively. Figure 4 shows the heat map of stage 1; the accuracy reaches 92%. Figure 5 shows the detection performance of Faster-RCNN in recognizing anatomical parts and foci; the mean value of average precision over all classes surpasses 82% and 90% for images under natural light without fluorescein sodium eye drops, and images under cobalt blue light or natural light with fluorescein sodium eye drops, respectively. The left image in Figure 5 is the performance for localizing objects in images without fluorescein sodium eye drops during stage 2, where I-VX represent the cornea and iris zone with keratitis, the focus of keratitis, the conjunctiva and sclera zone, the slit arc of the cornea, the slit arc of keratitis focus, the eyelid, the slit arc of the iris, the conjunctiva and sclera zone with hyperemia, the conjunctiva and sclera zone with edema, cornea and iris zone, pterygium, eyelash, pupil zone, the conjunctiva and sclera zone with hemorrhage, and the pupil zone with cataracts, respectively. The right image in Figure 5 presents the performance for localizing the objects in images with fluorescein sodium eye drops during stage 2, where I-VII represent the cornea and iris zone with keratitis, the focus of keratitis, the slit arc of the cornea, the slit arc of keratitis focus, the slit arc of the iris, the eyelid, and the eyelash, respectively. The statistical results of stage 2 are shown in Multimedia Appendix 1.

### Performance of Stages 3 and 4

**Figure 6:**
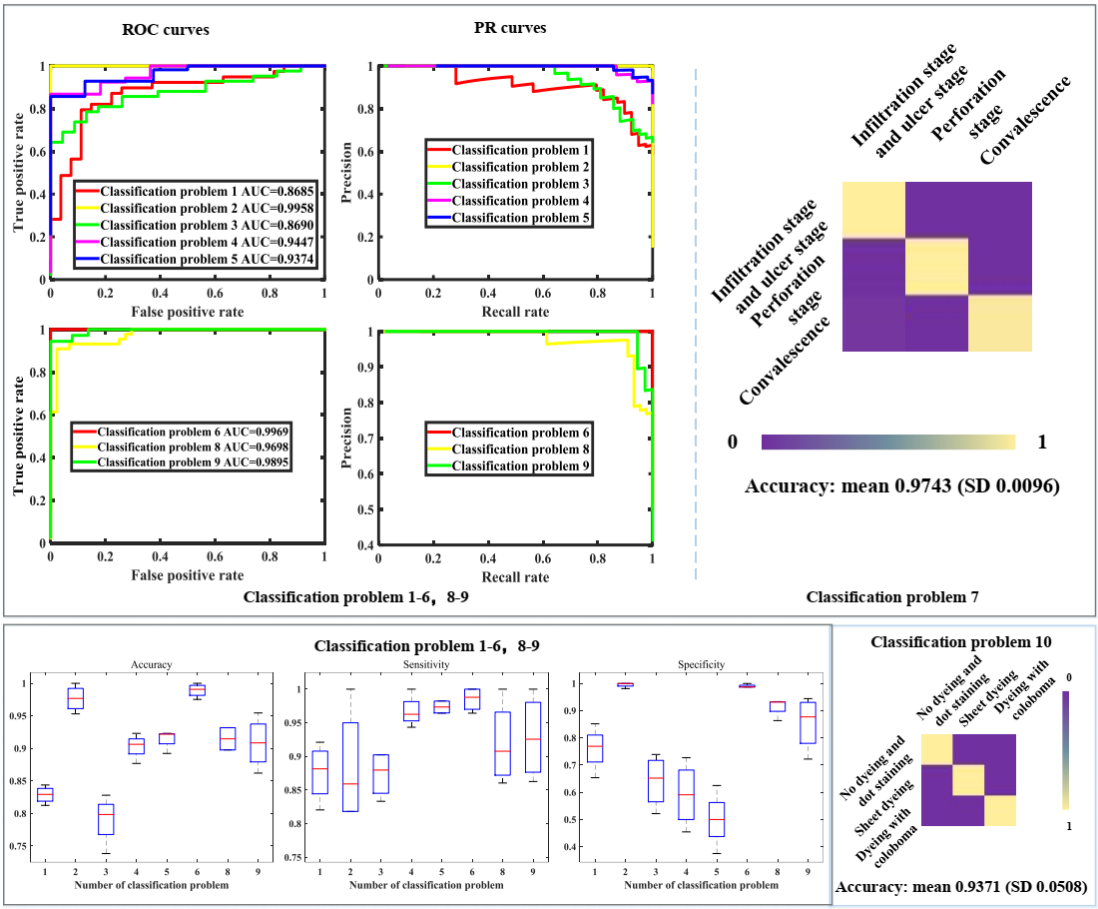
Performance of stage 3 and 4. PR: precision recall; ROC: receiver operating characteristics.

Stage 3 was decomposed into 10 classification problems, and the relevant results are shown in Figure 6, including the boxplots for the accuracy, specificity and sensitivity, ROC curve with the AUC, PR curve for all binary classification problems, and the heat maps with accuracy for all multiclass classification problems. Figure 6 also shows the classification performance of stage 4, which includes boxplot for the accuracy, sensitivity and specificity, ROC curve with the AUC value and PR curve. The only one classification problem addressed by stage 4 is whether a patient who suffers from pterygium needs surgery. In stage 2, the detection rate of some objects is low because Faster-RCNN cannot effectively detect some small objects. We will overcome this issue by adjusting the parameters of Faster-RCNN. In spite of this, stage 3 will not be affected by this drawback because the detection rate of the cornea and iris zone with keratitis and pterygium (the relevant anatomical parts and foci), which is involved with stage 3, is considerably high. In addition, the detection performance of the pupil zone, which is related to vision is also satisfactory. In stage 3, the specificity of classification problems 1, 3, 4, and 5 is slightly low; the application scene of this system is hospitals where doctors pay more attention to sensitivity than specificity. The result of all classification problems is satisfactory and acceptable. Furthermore, the performance of classification problems 1, 3, 4, and 5 can be improved with more samples under the circumstance of Web-based learning. The statistical results of stages 3 and 4 are shown in Multimedia Appendix 1.

### Performance of Stage 3 and 4 with Original Images

**Figure 7:**
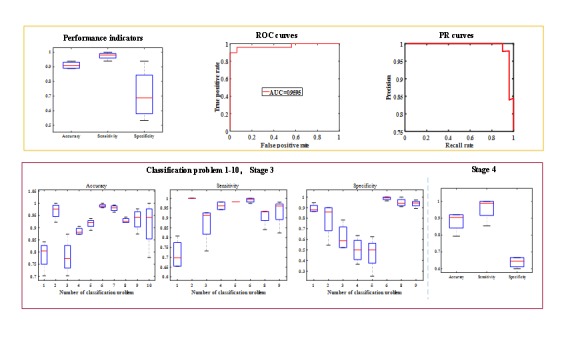
Performance of stage 3 and 4 with original images. PR: precision recall; ROC: receiver operating characteristics; AUC: area under the curve.

To study which anatomical parts are essential for automatic diagnostic, stages 3 and 4 were repeated with original medical images without processing; all parameters were same as the original parameters used in stages 3 and 4. The relevant results are shown in Figure 7. The classification performance close to that of the classification with anatomical parts and foci. In other words, the important parts, the cornea and iris zone with keratitis and pterygium, are essential for automatic diagnosis. The statistical results of stages 3 and 4 with original images are shown in Multimedia Appendix 1.

### Web-Based Automatic Diagnostic System

We applied Django framework [34] to develop a telemedical decision-making and automatic diagnosing system to facilitate doctors and patients; this system can analyze inputted medical images, show the diagnostic result as the working process of doctors, and provide treatment advice by producing an examination report. In addition, this telemedical system can finely analyze medical images and provide treatment advice with a diagnostic report (a PDF file) that includes treatment suggestion according to the analysis result and the consultation between a doctor and a patient. The format of the diagnostic report is shown in Multimedia Appendix 1. All diagnostic information can be shown to a doctor and a patient by storing into a database. Administrators and doctors can handle all information and contact patients conveniently. Furthermore, this system can be deployed in multiple hospitals and medical centers to screen common diseases and collect more medical data, which can be used to improve the diagnosis performance. The website is available in Multimedia Appendix 1.

## Discussion

In this study, we constructed an explainable artificial intelligence system for the automatic diagnosis of multiple ophthalmic diseases. This system carefully mimics the work flow of doctors so that reasons for specific diagnosis can be explained to doctors and patients with high performance. Besides, this system accelerates the application of telemedicine with the assistance of computer network and helps develop the health level and medical condition. Moreover, this system can be easily expanded to cover more diseases as long as the diagnostic processes of other diseases are simulated seamlessly. In addition, this system can help medical students to understand diagnosis and diseases. In the future, considerable progress can be made in this field. In this research, we did not consider a multilabel classification for those patients with multiple diseases. In the future, multiple-label classification can be adopted to make this system closer to real clinical circumstances. Moreover, because the bound box is not suitable for some anatomical parts, semantic segmentation can be applied in this system for segmenting medical images more accurately.

## Appendix

Multimedia Appendix 1 Relevant material.

## Abbreviations

AUC: area under receiver operating characteristics curve
CNN: Convolutional Neural Network
PR: precision recall
RCNN: region based convolutional neural network
Resnet: residual network
ROC: receiver operating characteristics
