# Supplementary material for: An Interpretable and Expandable Deep Learning Diagnostic System for Multiple Ocular Diseases: Qualitative Study
Source: J Med Internet Res. 2018 Nov 14;20(11):e11144. doi: 10.2196/11144 (PMC6301833; doi:10.2196/11144)
Supplement: Multimedia Appendix 1 [file jmir_v20i11e11144_app1.pdf]

Interpretable and Expandable Deep Learning Diagnosis System for Multiple Ocular Diseases: Elaborately Simulating Doctors' working

Kai Zhang[1, 2], Xiyang Liu[1, 3, 4], Fan Liu[3], Lin He[1], Lei Zhang[1], Yahan Yang[2], Wangting Li[2], Shuai Wang[3] , Lin Liu[1], Zhenzhen Liu[2], Xiaohang Wu[2], Haotian Lin[2§]

[1]School of Computer Science and Technology, Xidian University, Xi'an 710071, China;

[2]State Key Laboratory of Ophthalmology, Zhongshan Ophthalmic Center, Sun Yat-sen University, Guangzhou 510060, China;

[3]School of Software, Xidian University, Xi'an 710071, China;

[4]Institute of Software Engineering, Xidian University, Xi'an 710071, China;

[§]**Corresponding authors:**

Prof. Haotian Lin, Email: haot.lin@hotmail.com; Address: Zhongshan Ophthalmic Center, Xian Lie South Road 54#, Guangzhou, China, 510060. Telephone: +86-020-87330493; Fax: +86-020-87333271.

The format of AP, accuracy, sensitivity and specificity is mean ± standard derivation. The web-based automatic diagnostic system can be freely access with the url: http://114.67.37.252:80.

Supplementary Table 1. Statistical results of stage 2 (images under natural light without fluorescein sodium eye drops).

Eyelash	Conjunctiva and sclera zone with hemorrhage	Pupil zone with cataract
0.6395 ± 0.0096	0.9083 ± 0.0015	0.8398 ± 0.0462
Eyelid	Conjunctiva and sclera zone with edema	Conjunctiva and sclera zone
0.8008 ± 0.0367	0.7403 ± 0.0649	0.7823 ± 0.0093
Pupil zone	Slit arc of keratitis focus	Slit arc of cornea
0.8930 ± 0.0089	0.7228 ± 0.0997	0.8491 ± 0.0587
Slit arc of iris	Conjunctiva and sclera zone with hyperaemia	Cornea and iris zone
0.8175 ± 0.0344	0.6679 ± 0.0386	0.8609 ± 0.0528
Pterygium	Cornea and iris zone with keratitis	Focus of keratitis
0.9755 ± 0.0443	0.9975 ± 0.0016	0.7624 ± 0.0586

Supplementary Table 2. Statistical results of stage 2 (images under cobalt blue light or natural light with fluorescein sodium eye drops).

Cornea and iris zone with keratitis		Focus of keratitis	Slit arc of cornea
1 ± 0		0.6749 ± 0.0502	1 ± 0
Slit arc of keratitis focus	Slit arc of iris	Eyelid	Eyelash
0.8052 ± 0.1858	0.9659 ± 0.0682	0.9960 ± 0.0057	0.8578 ± 0.0060

Supplementary Table 3. Statistical results of stage 3.

No. of classification problem	Accuracy	Sensitivity	Specificity
1	0.8287 ± 0.0132	0.8768 ± 0.0433	0.7610 ± 0.0814
2	0.9767 ± 0.0201	0.8762 ± 0.0859	0.9953 ± 0.0095
3	0.7992 ± 0.0435	0.8738 ± 0.0344	0.6413 ± 0.0964
4	0.9032 ± 0.0192	0.9672 ± 0.0237	0.5909 ± 0.1174
5	0.9148 ± 0.0150	0.9735 ± 0.0102	0.5000 ± 0.1021
6	0.9892 ± 0.0105	0.9850 ± 0.0179	0.9904 ± 0.6604
8	0.9147 ± 0.0197	0.9190 ± 0.0616	0.9151 ± 0.0344
9	0.9084 ± 0.0390	0.9285 ± 0.0631	0.8553 ± 0.0996

Supplementary Table 4. Statistical results of stage 4.

Disease	Pterygium
Accuracy	0.9107 ± 0.0241
Sensitivity	0.9745 ± 0.0257
Specificity	0.7115 ± 0.1749

Supplementary Table 5. Statistical results of stage 3 with original images.

No. of classification problem	Accuracy	Sensitivity	Specificity
1	0.7884 ± 0.0598	0.8930 ± 0.0389	0.7140 ± 0.0747
2	0.9647 ± 0.0295	0.7909 ± 0.1681	1 ± 0
3	0.7807 ± 0.0702	0.8715 ± 0.0938	0.6196 ± 0.1249
4	0.8849 ± 0.0152	0.9616 ± 0.0223	0.4997 ± 0.1173
5	0.9169 ± 0.0203	0.9819 ± 0	0.4688 ± 0.1573
6	0.9892 ± 0.0078	0.9881 ± 0.0168	0.9904 ± 0.0123
7	0.9804 ± 0.0140	——	——
8	0.9291 ± 0.0113	0.9482 ± 0.0390	0.9094 ± 0.0457
9	0.9341 ± 0.0444	0.9382 ± 0.0335	0.9314 ± 0.0725
10	0.9154 ± 0.0962	——	——

Supplementary Table 6. Statistical results of stage 4 with original images.

Disease	Pterygium
Accuracy	0.8813 ± 0.0602
Sensitivity	0.9584 ± 0.0701
Specificit y	0.6396 ± 0.0329

Supplementary Figure 1. Format of diagnostic report (PDF).

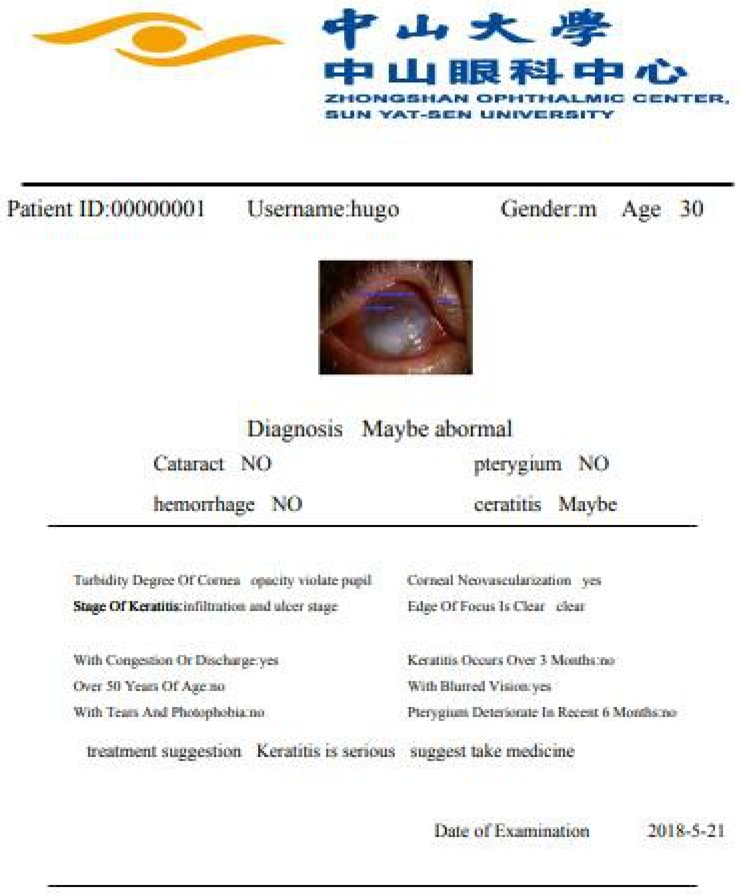

中山大學
中山眼科中心
ZHONGSHAN OPHTHALMIC CENTER,
SUN YAT-SEN UNIVERSITY

Patient ID:00000001 Username:hugo Gender:m Age 30

Diagnosis Maybe abormal

Cataract NO pterygium NO

hemorrhage NO ceratitis Maybe

Turbidity Degree Of Cornea opacity violate pupil

Stage Of Keratitis:infiltration and ulcer stage

Corneal Neovascularization yes

Edge Of Focus Is Clear clear

With Congestion Or Discharge:yes

Over 50 Years Of Age:no

With Tears And Photophobia:no

Keratitis Occurs Over 3 Months:no

With Blurred Vision:yes

Pterygium Deteriorate In Recent 6 Months:no

treatment suggestion Keratitis is serious suggest take medicine

Date of Examination 2018-5-21
